# Dosimetric and radiobiological evaluations as well as technical aspects of left breast treatment employing DIBH with SGRT in radiotherapy with IMRT step and shoot, IMRT Dynamic MLC and VMAT

**DOI:** 10.1002/acm2.70761

**Published:** 2026-08-25

**Authors:** Julien Rolland, Stéphane Payan, Pierre Fau, Hugues Mailleux, Agnès Tallet

**Affiliations:** ^1^ Department of Medical Physics Centre Hospitalier InterCommunal des Alpes du Sud Gap France; ^2^ Department of Medical Physics Institut Paoli Calmettes Marseille France; ^3^ Department of Radiotherapy Institut Paoli Calmettes Marseille France

**Keywords:** DIBH, DMLC‐IMRT, IMRT, left breast, NTCP, SCCP, SGRT, SS‐IMRT, TCP, VMAT

## Abstract

**Background:**

Treatment of the left breast using deep inspiration breath hold (DIBH), combined with surface guided radiotherapy (SGRT), has emerged as the standard for reducing the dose to the heart, the left anterior descending coronary artery (LAD), and the ipsilateral lung. Since this treatment is administered during breath hold (BH) phases, it is beneficial to limit the duration of delivery to improve patient comfort. Furthermore, the SGRT signal can be disrupted when the gantry passes in front of the left SGRT camera during the delivery with volumetric modulated arc therapy (VMAT), which complicates the treatment process. In contrast, using a simpler treatment plan with two tangential beams with intensity modulated radiation therapy (IMRT) helps avoid this difficulty. For image guided radiotherapy (IGRT) as well, monitoring becomes more complex with the addition of the imaging panel and the X‐ray tube for the SGRT signal.

**Purpose:**

This study compares dosimetric and radiobiological differences between two methods in IMRT step and shoot (SS‐IMRT) or dynamic multileaf collimator (DMLC‐IMRT) using two tangential beams and VMAT with two arcs.

**Methods:**

Twenty left breasts were planned using SS‐IMRT, DMLC‐IMRT and VMAT. The comparison is based on dosimetric criteria at target (D_95%_, homogeneity index (HI), conformity index (CI)) and doses to organs at risk (OARs). Radiobiological parameters (tumor control probability (TCP), normal tissue complication probability (NTCP) and second cancer complication probability (SCCP)) and plan data such as MLC modulation, QA results, and delivery time were also compared. A Wilcoxon signed‐rank test was performed between each method.

**Results:**

The results are listed for SS‐IMRT, DMLC‐IMRT and VMAT, respectively. For the PTV: D95% 37.5 Gy, 38.3 Gy, 38.4 Gy (*p <* 0.05 DMLC‐IMRT & VMAT vs SS‐IMRT); CI 0.74, 0.76, 0.83 (*p <* 0.05); HI 0.15, 0.11, 0.11 (p > 0.05). TCP: 91.2%, 92.2% and 92.9% (*p <* 0.05). For OARs; heart: D_mean_ 0.8 Gy, 1.0 Gy, 1.2 Gy (*p <* 0.05); LAD: V_10Gy_ 0.5%, 0.1%, 0.0% (*p <* 0.05 except DMLC‐IMRT vs VMAT); ipsilateral lung: D_mean_ 5.0 Gy, 4.8 Gy, 5.7 Gy (*p <* 0.05), V_5Gy_ 19.4 %, 18.6 %, 25.7%. NTCP pericarditis: 0% for all techniques. NTCP pneumonitis: 4.1%, 3.7%, 2.2%; contralateral lung: D_mean_ 0.3 Gy, 0.5 Gy, and 0.9 Gy; contralateral breast: D_mean_: 0.6 Gy, 0.7 Gy and 1.2 Gy, V_3Gy_ 0.6%, 0.4%, 1.7% (*p <* 0.05), SCCP: 0.4%, 0.5%, 0.9%. Plans data: more modulated in DMLC‐IMRT but QA results remained satisfactory. The average delivery times are 95.4s, 81.6s, 141.7s.

**Conclusions:**

In most cases, choosing DMLC‐IMRT will result in a plan similar to VMAT and superior to SS‐IMRT. Compared to SS‐IMRT, DMLC‐IMRT delivers a slightly higher dose to contralateral OARs, with a slight increase in the risk of radiation‐induced cancer, but lower than VMAT. The main advantage is that it eliminates camera occlusion issues, which can lead to erroneous SGRT signals during treatment and IGRT verification. DMLC‐IMRT reduces treatment time by an average of 42% compared to VMAT, which will remain the solution for the most complex morphologies.

## 1 INTRODUCTION

Breast cancer is among the most common cancers worldwide, and radiotherapy is widely used for its treatment. A major concern, especially for left‐sided breast cancer, is radiation exposure to the heart.^1^ To reduce this risk, deep inspiration breath hold (DIBH) has become standard with the development of surface guided radiotherapy (SGRT).^2^

Breast radiotherapy initially relied on 3D conformal radiation therapy (3DCRT) with tangential fields and wedge filters to compensate for tissue thickness. It later evolved to field‐in‐field planning, which improved dose homogeneity, and to intensity modulated radiation therapy (IMRT), where two tangential IMRT beams (tIMRT) reduce dose to organs at risk (OARs) compared with 3DCRT. Depending on patient anatomy and OAR sparing, volumetric modulated arc therapy (VMAT) may be preferable.^3^ Studies show that VMAT improves target conformity but spreads a more diffuse dose elsewhere, while multi‐beam IMRT can achieve similar results.^4^, ^5^, ^6^, ^7^, ^8^, ^9^, ^10^, ^11^, ^12^ Overall, IMRT and VMAT offer different trade‐offs, and plan quality appears similar between full arc (generally arc of 200° to 240°) conformal VMAT (cVMAT) and partial‐arc (generally arc of 50° to 60°) tangential VMAT (tVMAT). A recent European study^13^ reported the diversity of techniques, VMAT for the left breast alone was used in fewer than 20% of cases. VMAT is the treatment of choice for complex cases, particularly those requiring irradiation of lymph node regions.

In SGRT, the patient surface from the planning scanner is used as a reference. Three cameras capture the surface within a defined region of interest (ROI) and compare it with the real‐time surface. If deviations exceed user‐defined tolerances, the beam is interrupted either automatically, if a beam‐on/beam‐hold interface is connected between the linear accelerator and the SGRT system, or manually by the radiotherapy technologists (RTTs). During DIBH, patients hold a deep inspiration for several seconds, often repeatedly during a session. Faster delivery of treatment shortens apnea time. SGRT reduces localization uncertainty by improving position reproducibility, limiting internal target motion, and monitoring patient motion and breathing during DIBH, thereby increasing treatment accuracy.

The passage of the gantry^14^, ^15^ in front of an SGRT camera disrupts surface reconstruction, leading to a mismatch with the initial reference surface. Generally, this is avoided by defining a sufficiently large ​​ROI^16^ (from the left breast to the middle of the right breast) to maintain an adequate signal. Obstruction of the left camera affects left breast treatments for gantry angles between 20° and 60°.^14^ Monitoring is more challenging during cone‐beam computed tomography (CBCT) because the kilovoltage (kV) imaging panel and X‐ray tube, in addition to the gantry and the megavoltage (MV) imaging panel, interfere with the SGRT signal. In contrast, during MV imaging for tIMRT, the constraints are less severe because only the gantry and the MV imaging panel obstruct the SGRT cameras, allowing adequate SGRT signal acquisition. In an internal study, optimization of an extended region of interest (ROI) reduced gantry‐related camera obstruction to less than 5% of treatment sessions. Depending on the system, the monitoring report may show a transient 1–2 mm deviation lasting approximately 20 seconds, while rotational parameters remain unaffected. This is within the 3 mm lateral/longitudinal tolerance but can exceed the 1 mm vertical tolerance used for DIBH. In free breathing (FB), RTTs can distinguish patient motion from camera obstruction with the gantry at 0°, the values remain out of tolerance, the patient has moved and RTTs reposition the patient; otherwise, the gantry is the cause, and the beam hold is temporarily disabled and then re‐enabled once the gantry has passed the obstruction sector. A dedicated procedure/decision tree secures this workflow. In DIBH, it is more problematic because the patient is asked to hold their breath several times, and it will not be possible to complete the treatment if a camera obstruction is present. Therefore, a first‐session gantry pass test is performed to optimize the ROI if possible. If it fails, an IMRT/VMAT plan avoiding these angles is required, delaying treatment start and impacting workflow. In DIBH, a 1–2 mm vertical bias can trigger out of tolerance feedback, prompting patients to overcorrect their apnea for 20 seconds. If lateral/longitudinal values are affected, RTTs must enter the room to check positioning (gantry at 0°, patient in apnea) and test with the gantry at the correct position, adding minutes and increasing patient stress. This occurred only with VMAT and in < 5% of sessions, raising the question of whether cVMAT is justified or whether tIMRT could be used instead.

The use of VMAT or IMRT with beam arrangements that avoid these angles may be beneficial. The literature shows that tVMAT does not compromise plan quality. This study adds an additional dimension by introducing delivery time as a comparison criterion. Based on our dose results, several years ago, tVMAT were abandoned in favor of cVMAT with two arcs ranging from 170° to 300°, when tIMRT is not sufficient. The average dose to the ipsilateral lung was approximately 1 Gy higher, whereas the doses to the contralateral OARs was similar. This discrepancy stems from our institution's choice of isocenter position, which compromises the quality of the tVMAT plans. Partial arcs have been implemented when no beam hold system is available. A partial arc is delivered over a short period corresponding to a single apnea phase. IMRT uses two multileaf collimator (MLC) sequencing methods. In step and shoot IMRT (SS‐IMRT), the leaves move into position between irradiation segments, with the beam turning on and off between each segment. Dynamic MLC IMRT (DMLC‐IMRT), or sliding window IMRT, delivers radiation continuously while the MLC leaves move to create the field. In VMAT, the gantry rotates continuously around the patient while gantry speed, MLC shape, and dose rate are controlled simultaneously.

Although dosimetric comparisons have previously been reported, showing greater dose homogeneity with VMAT and improved sparing of lateral OARs with IMRT, this study establishes the relationship between the dosimetric plan (including radiobiological data) and its delivery over the course of treatment. It also evaluates the impact of the constraint caused by different types of image guided radiotherapy (IGRT) and potential camera obstructions, which, although infrequent after ROI optimization, may still occur during VMAT delivery and pose challenges for RTTs.

## 2 MATERIALS AND METHODS

### 2.1 Patient selection

Twenty consecutive plans were replanned for tIMRT and cVMAT and anonymized.

### 2.2 Simulation and contour

Computed tomography (CT) scans were acquired with patients positioned supine on an inclined plane (Civco Medical Instruments Co., Inc., Orange, IA, USA) using a Siemens SOMATOM go.Open Pro scanner (Siemens Healthineers, Erlangen, Germany). The clinical target volume (CTV) was delineated according to ESTRO guidelines, and a 5 mm margin was added to create the planning target volume (PTV), excluding the 5 mm skin layer beneath the surface. OARs were the heart, left anterior descending coronary artery (LAD), ipsilateral and contralateral lungs, and contralateral breast.

### 2.3 Prescription, dose and delivery

A total dose of 40.05 Gy was delivered in 15 fractions of 2.67 Gy. Target and OAR doses had to meet our institutional constraints (Table 1). The D_95%, PTV_ constraint was flexible, allowing a small underdosed PTV volume if the CTV remained covered, as confirmed by the physician during plan review. All plans were normalized to a median PTV dose of 40.05 Gy (D_50%, PTV_ = 40.05 Gy) and optimized to minimize OAR dose while maintaining adequate target coverage. SGRT with AlignRT (VisionRT, London, UK) was used for setup optimization and DIBH monitoring, with automatic beam on and beam hold controlled via the Elekta Response module.

**TABLE 1:** Left breast planning acceptance criteria and data analyzed.

Volumes		Constraints	Data analyzed
PTV	D_95%_ (Gy)	dose distribution display	D_98%_ (Gy), D_95%_ (Gy), V_95%_ (%), D_2%_ (Gy), HI, CI
	D_2%_ (Gy)	≤ 105% prescribed dose	
CTV	D_95%_ (Gy)	≥ 95% prescribed dose	D_98%_ (Gy), D_95%_ (Gy), D_2%_ (Gy)
	D_2%_ (Gy)	≤ 105% prescribed dose	
Heart	D_mean_ (Gy)	≤ 3 Gy	D_mean_ (Gy), D_2%_ (Gy), V_3Gy_ (%)
LAD	V_10Gy_ (%)	≤ 2 %	D_mean_ (Gy), D_2%_ (Gy), V_10Gy_ (%)
Ipsilateral lung	D_mean_ (Gy)	≤ 15 Gy	D_mean_ (Gy), V_5Gy_ (%), V_17Gy_ (%),V_30Gy_ (%)
	V_5Gy_ (%)	≤ 50%	
	V_17Gy_ (%)	≤ 14%	
Contralateral lung	D_mean_ (Gy)	≤ 1 Gy	D_mean_ (Gy)
Contralateral breast	V_3Gy_ (Gy)	≤ 10%	D_mean_ (Gy), D_2%_ (Gy), V_3Gy_ (%)
Other		NA	V_isodose5Gy_ (cm^3^), V_isodose20Gy_ (cm^3^)

Abbreviations:; CI, conformal index; CTV, clinical target volume; Dmean, median dose; Dx, dose to x% of the volume; HI, homogeneity index; LAD, left anterior descending coronary artery; PTV, planning target volume; Vx, % volume receiving x Gy.

### 2.4 Treatment plans

All plans were optimized on RayStation treatment planning system (TPS) in version V.10A (RaySearch Laboratories AB, Stockholm, Sweden) by the same user to avoid potential bias from varying planner expertise or planning differences. The only remaining concern is a potential systematic bias that might be introduced by a single planner; however, this will have less of an impact on the discrepancies between the plans than a random bias caused by multiple planners. Treatments were delivered using 6 MV photons on an Elekta Infinity accelerator (ELEKTA, Stockholm, Sweden) with an Agility MLC (80 pairs of 5 mm thick leaves at the isocenter). A 3 mm dose grid was applied.

The isocenter is positioned at the patient's center to ensure the PTV is within a 20 cm radius, allowing the MLC to treat a slightly larger volume than the PTV. Table height is adjusted to facilitate collision free‐CBCT imaging with the linac as well as during treatment.

For DMLC‐IMRT, the beam remains constant while the MLC navigates across the treatment aperture at varying rates to deliver a continuous fluence pattern. In contrast, SS‐IMRT involves setting the MLC to a series of discrete aperture shapes and delivering the beam only when the leaves are stationary at each position. In SS‐IMRT, users can specify the minimum segment size, the number of segments, and the minimum number of monitor units (MU) per segment. Our institution has limited the segment size to 3 cm^2^ and the minimum number of MU to 4, with a total of 12 different MLC segment shapes. In dynamic mode, the only adjustable parameter is the number of control points (CP) per beam. As DMLC‐IMRT is a new implementation in our institution, plans with 5, 10, and 20 CPs were created. Typically, tangential beams have a width of approximately 10 cm in the leaf movement direction. Consequently, 20 CPs correspond to roughly 2 CPs per cm, 10 CPs to 1 CP per cm, and 5 CPs to 1 CP every 2 cm. The results for 5 CPs are excluded due to unsatisfactory dosimetric outcomes. Therefore, 10 and 20 CPs (designated as DMLC10‐IMRT and DMLC20‐IMRT) were utilized to evaluate the impact of CP in DMLC. For SS/DMLC‐IMRT, the collimator angle was set to 0°. Tangential beam gantry angles were selected using the beam's eye view (BEV) to minimize exposure to the LAD, heart, contralateral breast, and, when possible, the left lung. VMAT plans consisted of two arcs from 170° to 300° (cVMAT), delivered clockwise and counterclockwise. The dual arc approach optimizes a single set of fluence profiles across both opposing arcs, preserving fluence information, reducing leaf motion and OAR exposure, and improving organ sparing. All arcs used one CP every 3° of gantry rotation and a collimator angle of 15°.

To account for breast motion and swelling during a fraction, particularly under FB conditions, two methods were used. It was also decided to perform this procedure with DIBH, as SGRT allows average surface area verification, although minor swelling within SGRT tolerances (1 mm vertical, 3 mm lateral and longitudinal) may still go undetected. For VMAT, a 1 cm virtual bolus was applied during planning and removed after optimization.^17^ For IMRT, robust optimization simulated 1.5 cm leftward and anterior PTV shifts to model setup errors from slight breast displacement or swelling, allowing the TPS to adapt the MLC to cover tissue extending beyond the breast on CT. Supplementary Material Figure S1 illustrates the cVMAT and tIMRT beam arrangement.

### 2.5 Plan comparison

#### 2.5.1 Dosimetric parameters

For all plans, homogeneity index (HI) and conformity index (CI) for the PTV were evaluated, as well as dose or coverage values D_98%_, D_95%_, D_2%_. D_98%_ and D_95%_ are the dose delivered to 98% or 95% of the PTV volume, respectively. D_2%_ represents the maximum dose delivered in 2% of the PTV volume. The values corresponding to Table 1 were reported.

HI is calculated using the following formula:

(1) $$\mathrm{HI}\;=\frac{D_{2\%}-D_{98\%}}{D_{50\%}}\;$$

D_50%_ is the median dose of the PTV. An optimal value is 0 and the HI value varies between 0 and 1.

CI defined by Paddick is:

(2) $$\mathrm{CI}\;=\frac{V_{T_{\mathrm{pres}}}^{2}}{\mathrm{TV}\times V_{\mathrm{pres}}}\;$$

Where $V_{T_{\mathrm{pres}}}$ is the volume of the PTV that receives at least the prescribed dose. TV is the volume of the PTV. $V_{\mathrm{pres}}$ is the volume that receives at least the prescribed dose. An optimal value is 1 and the CI value varies between 0 and 1.

#### 2.5.2 Radiobiological parameters

The Niemierko's formalism and the Lyman‐Kutcher‐Burman (LKB) model were used respectively to calculate the tumor control probability (TCP) and the normal tissue complication probability (NTCP). NTCP was calculated for pericarditis (heart) and pneumonitis (lungs).

The risk of radiation‐induced secondary cancer was evaluated using the Schneider model, which estimates the second cancer complication probability (SCCP) for the contralateral breast, the contralateral lung and both lungs. This model accounts for the non‐linear dose‐response relationship of carcinogenesis and is particularly sensitive to low dose exposure.

Parameters for TCP, NTCP and SCCP were given in Supplementary Table S1. Radiobiological values are calculated from DVHs exported from RayStation with Python in version 3.13.

### 2.6 Plan parameters

#### 2.6.1 Quality control

Measurements were performed with an ArcCheck diode array (Sun Nuclear Corporation, Melbourne, FL, USA). Gamma analysis used 3%/3 mm criteria, global dose normalization, and a 10% low‐dose threshold. A Gamma Passing Rate (GPR) of 95% was used as the acceptance criterion: plans above this threshold were considered acceptable, and those below it unacceptable.

#### 2.6.2 MLC complexity metrics

The complexity metrics employed in this study aim to describe the general movement and aperture shape of treatments involving MLC:

- The total number of MUs.
- The modulation complexity score (MCS) (for IMRT only), originally defined by McNiven et al., is used to quantify the complexity of CP and the overall complexity of the beam. Lower MCS values indicate more intricate plans. The MCS ranges from 0 to 1. A value of MCS equal to 1 indicates no modulation, such as a beam with a fixed rectangular aperture with no moving leaves during the beam. As modulation increases, the MCS value decreases.

### 2.7 Statistical analysis

Differences between treatment techniques were assessed using a paired Wilcoxon signed‐rank test. All patient data were included in the analysis, with no exclusions. A *p*‐value < 0.05 was considered statistically significant. The following comparisons were conducted: SS‐IMRT with DMLC20‐IMRT, SS‐IMRT with VMAT, DMLC20‐IMRT with DMLC10‐IMRT and DMLC20‐IMRT with VMAT.

## 3 RESULTS

### 3.1 Dose analysis

**FIGURE 1:**
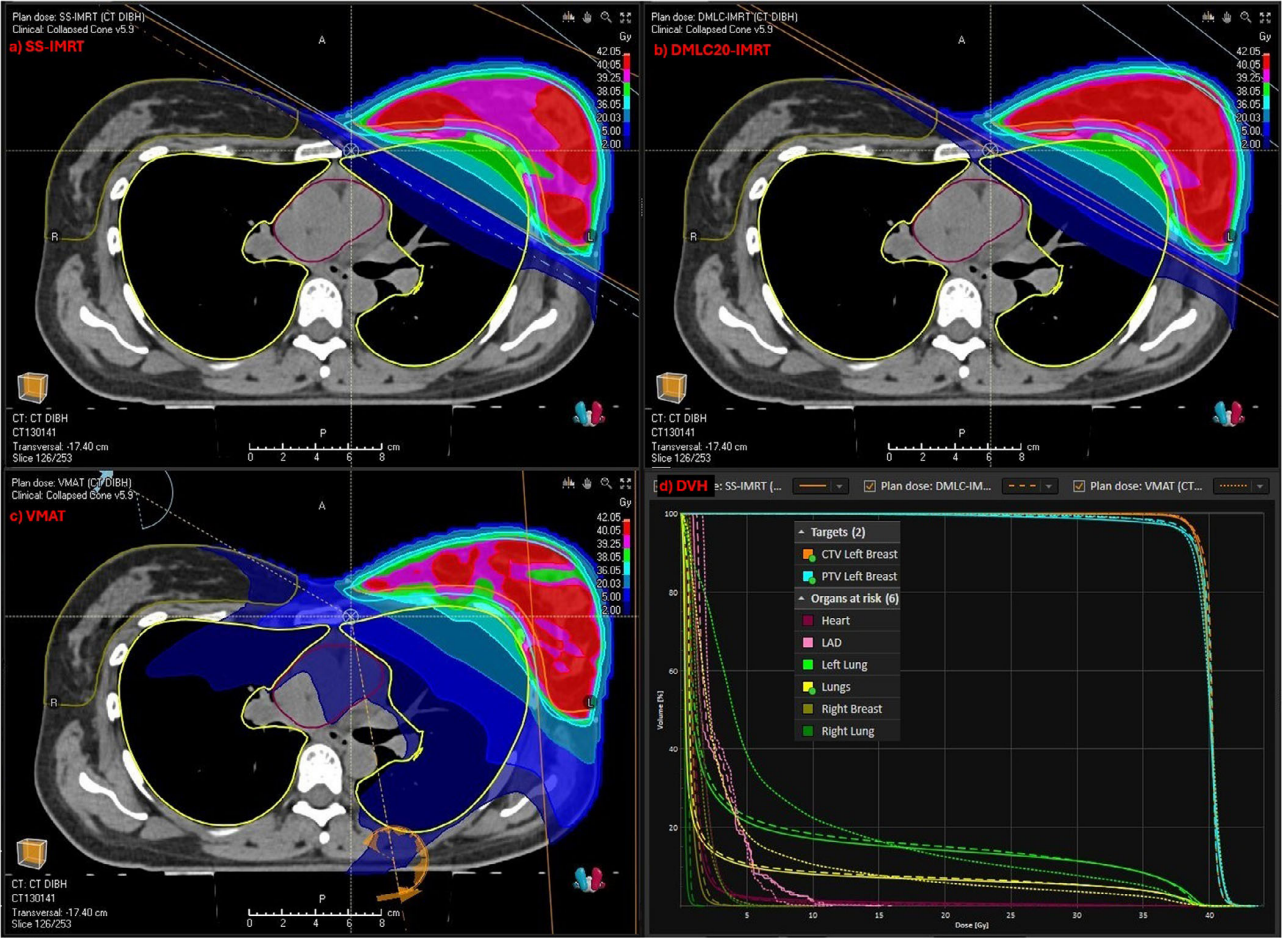
Dose distributions in 3 axial plans for an atypical morphology (17 cm breast diameter, LAD 4 mm from the tangential field edges, and 4 cm of left lung visible on BEV) for: a) SS‐IMRT; b) DMLC20‐IMRT; c) VMAT; d) DVH. Visible and contoured volumes are: PTV in light blue, CTV in orange, LAD in pink, heart in purple, left lung in light green, right lung in dark green, right breast in kaki green.

**FIGURE 2:**
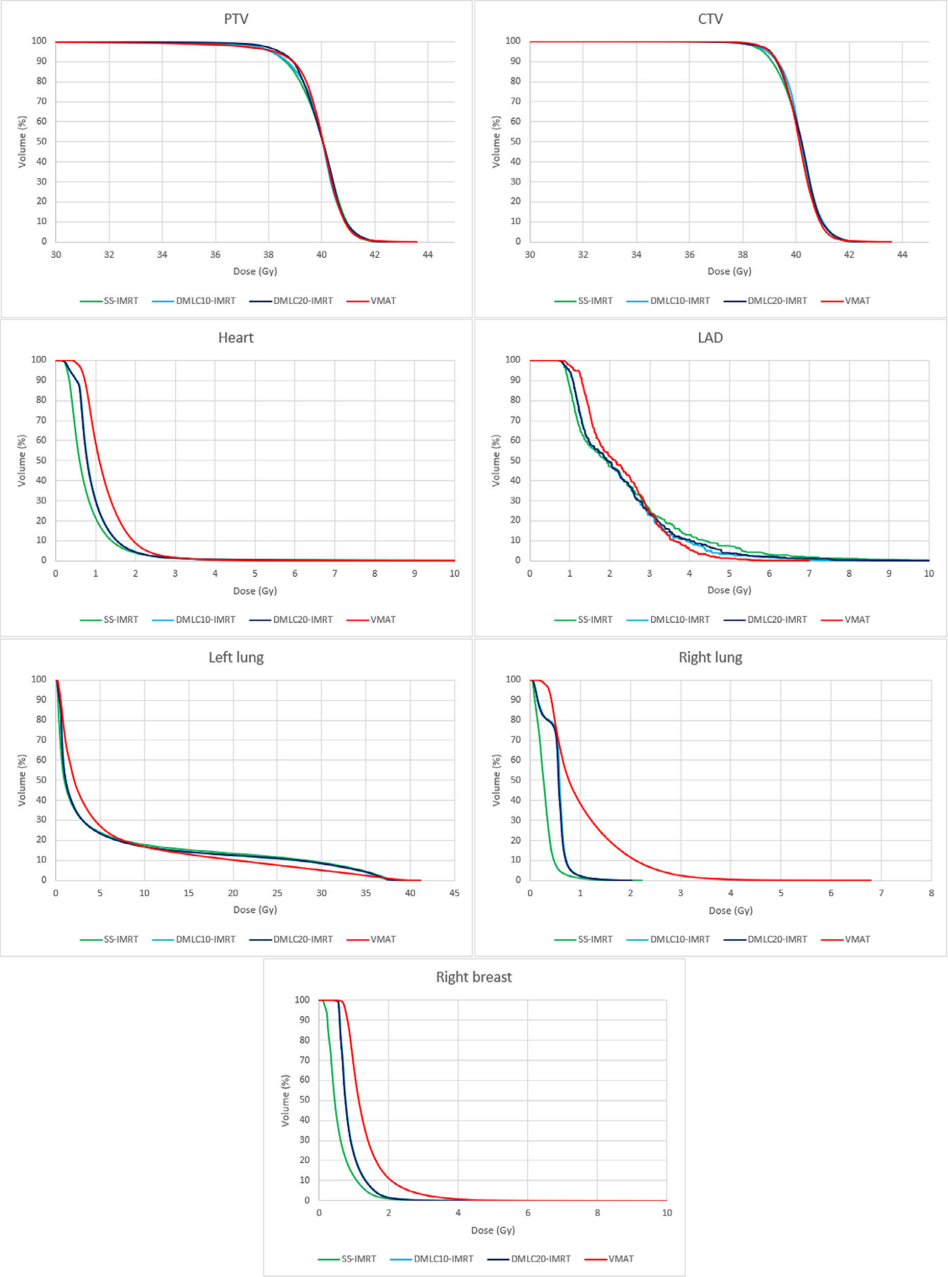
Dose volume histogram for a left breast treatment for PTV, CTV, heart, LAD, left and right lung and right breast for all techniques: SS‐IMRT in green, DMLC10‐IMRT in light blue, DMLC20‐IMRT in dark blue and VMAT in red.

Figure 1 and Figure 2 show, respectively, the dose distributions in three transversal slices for an atypical morphology and a DVH for a classical patient. The results for target volume and OARs are given in Table 2. A typical dose distribution in 3 axial planes for a classical patient and all methodologies is available in Supplementary Figure S2.

**TABLE 2:** Summary of the quantitative analysis of the dose volume histograms and radiobiological parameters of target volumes and OARs for all techniques.

		SS‐IMRT			DMLC10‐IMRT			DMLC20‐IMRT			VMAT			*p*‐value	*p*‐value	*p*‐value	*p*‐value
		Mean	±	SD	Mean	±	SD	Mean	±	SD	Mean	±	SD	DMLC20 vs DMLC10	DMLC20 vs SS	DMLC20 vs VMAT	VMAT vs SS
PTV	D_98%_ (Gy)	35.9	±	1.8	36.8	±	1.2	**37.0**	±	1.3	37.0	±	1.0	***0.006***	***0.001***	0.956	***0.001***
	D_95%_ (Gy)	37.5	±	1.5	38.1	±	0.3	38.3	±	0.2	**38.4**	±	0.4	***<0.001***	***0.001***	0.218	***<0.001***
	D_2%_ (Gy)	41.8	±	0.3	41.8	±	0.3	41.6	±	0.3	**41.5**	±	0.3	***<0.001***	***0.013***	0.411	***<0.001***
	V_95%_ (%)	94.3	±	1.8	95.6	±	1.3	96.3	±	1.0	**96.6**	±	1.2	***<0.001***	***<0.001***	0.571	***<0.001***
	CI	0.74	±	0.05	0.76	±	0.05	0.77	±	0.05	**0.83**	±	0.06	***<0.001***	***<0.001***	***<0.001***	***<0.001***
	HI	0.15	±	0.05	0.12	±	0.03	0.11	±	0.03	**0.11**	±	0.03	***<0.001***	***<0.001***	0.756	***0.001***
	TCP (%)	91.2	±	2.6	91.5	±	1.9	92.2	±	1.3	**92.9**	±	0.6	***0.001***	***0.004***	***0.002***	**<0.001**
CTV	D_98%_ (Gy)	38.2	±	0.4	38.3	±	0.3	38.4	±	0.2	**38.6**	±	0.3	***<0.001***	***0.004***	***0.006***	***<0.001***
	D_95%_ (Gy)	38.7	±	0.2	38.8	±	0.2	38.9	±	0.1	**39.1**	±	0.2	***<0.001***	***0.003***	***0.033***	***<0.001***
	D_2%_ (Gy)	41.9	±	0.3	41.7	±	0.3	41.6	±	0.4	**41.5**	±	0.3	***<0.001***	***0.004***	0.723	***<0.001***
	TCP (%)	93.4	±	0.3	93.4	±	0.2	93.5	±	0.1	**93.5**	±	<0.1	0.295	0.507	0.277	0.422
Heart	D_mean_ (Gy)	**0.8**	±	0.2	1.0	±	0.2	1.0	±	0.2	1.2	±	0.2	0.071	***<0.001***	***<0.001***	***<0.001***
	D_2%_ (Gy)	2.9	±	1.2	2.7	±	0.7	**2.7**	±	0.7	2.9	±	0.9	***0.045***	0.614	0.052	0.277
	NTCP (%)	0.0			0.0			0.0			0.0			N/A	N/A	N/A	N/A
LAD	D_mean_ (Gy)	2.2	±	0.6	2.1	±	0.5	**2.1**	±	0.5	2.2	±	0.5	0.155	0.926	0.107	0.575
	V_10Gy_ (%)	0.5	±	1.0	0.1	±	0.3	0.1	±	0.2	**<0.1**	±	0.1	0.400	***0.004***	0.059	***0.004***
	D_2%_ (Gy)	6.0	±	2.8	4.8	±	1.7	5.0	±	1.9	**4.4**	±	1.6	0.179	***0.002***	0.061	***0.001***
Ipsilateral lung	D_mean_ (Gy)	5.0	±	0.9	4.9	±	1.0	**4.8**	±	1.0	5.7	±	0.8	***<0.001***	***0.026***	***<0.001***	***<0.001***
	V_5Gy_ (%)	19.4	±	3.1	18.8	±	3.3	**18.6**	±	3.3	25.7	±	4.1	***<0.001***	***0.001***	***<0.001***	***<0.001***
	V_17Gy_ (%)	11.2	±	2.7	10.4	±	2.7	**10.2**	±	2.8	10.5	±	2.3	***<0.001***	***<0.001***	0.261	0.133
	V_30Gy_ (%)	7.0	±	2.2	6.6	±	2.3	6.5	±	2.3	**4.5**	±	1.5	***0.031***	***0.002***	***<0.001***	***<0.001***
	NTCP (%)	4.1	±	2.4	3.7	±	2.5	3.7	±	2.5	**2.2**	±	1.5	***0.001***	***0.001***	***<0.001***	***<0.001***
Contralateral lung	D_mean_ (Gy)	**0.3**	±	<0.1	0.5	±	0.1	0.5	±	0.1	0.9	±	0.3	0.162	***<0.001***	***<0.001***	***<0.001***
	SCCP (%)	**0.4**	±	<0.1	0.6	±	0.1	0.6	±	0.1	1.1	±	0.3	0.058	***<0.001***	***<0.001***	***<0.001***
Lungs	SCCP (%)	**1.6**	±	0.2	1.8	±	0.2	1.8	±	0.2	2.6	±	0.3	***0.011***	***<0.001***	***<0.001***	***<0.001***
Contralateral breast	D_mean_ (Gy)	**0.6**	±	0.2	0.7	±	0.2	0.7	±	0.2	1.2	±	0.2	0.071	***<0.001***	***<0.001***	***<0.001***
	V_3Gy_ (%)	0.6	±	1.1	0.4	±	0.8	**0.4**	±	0.8	1.7	±	2.3	***0.004***	0.118	***<0.001***	***0.002***
	D_2%_ (Gy)	2.0	±	1.4	1.8	±	0.7	**1.8**	±	0.7	2.9	±	1.3	***0.008***	0.596	***<0.001***	***0.003***
	SCCP (%)	**0.4**	±	0.1	0.6	±	0.1	0.5	±	0.1	0.9	±	0.2	0.173	**<0.001**	***<0.001***	***<0.001***
V_isodose20Gy_	(cm^3^)	1486.0	±	394.2	1435.5	±	378.9	**1420.9**	±	379.7	1494.9	±	369.1	***<0.001***	***<0.001***	***<0.001***	***<0.001***
V_isodose5Gy_	(cm^3^)	1989.6	±	500.8	1935.7	±	497.1	**1916.6**	±	496.4	2754.7	±	647.8	***<0.001***	***<0.001***	***<0.001***	0.596

*Note*: all available patient data were included in the paired Wilcoxon signed‐rank test, and no observations were excluded from the analysis. The optimal average values are highlighted in bold. *p*‐values less than 0.05 are highlighted in bold italics. A *p*‐value of less than 0.05 is considered to be significant.

Abbreviations: DMLC10 or DMLC20, dynamic multi leaf collimator with 10 or 20 controls points; IMRT, intensity modulation radiation therapy; NCTP, normal tissue complication probability; SCCP, second cancer complication probability; SD, standard deviation; SS, step and shoot; TCP, tumor control probability; VMAT, volumetric modulated arc therapy; N/A, not applicable.

For greater clarity, the results are given by comparing: the 2 dynamic techniques (DMLC10 vs DMLC20‐IMRT), SS‐IMRT vs DMLC20‐IMRT, SS‐IMRT vs VMAT and DMLC20‐IMRT vs VMAT.

The results for DMLC are statistically significant for PTV and CTV for D_98%_, D_95%_, D_2%_, HI, CI, isodose 5 and 20 Gy in favor of DMLC20‐IMRT. Dose gains were less than 0.2 Gy. CI and HI improvements were 1.5% and 7.9%, respectively. The differences in isodoses were less than 1%. For OARs, there were slight gains of 0.2 Gy at best for DMLC20‐IMRT.

Between DMLC20‐IMRT and SS‐IMRT, the results are statistically significant for D_98%,_ D_95%_ and D_2%_ with a gain of 1.1 Gy, 0.8 Gy and 0.2 Gy with DMLC20‐IMRT. Conformity improved by 4.2%, and the 20 Gy and 5 Gy isodoses decreased by 4.6% and 3.8%. In terms of OARs, the V_10Gy_ and D_2%_ of the LAD were reduced by 0.4% and 1.0 Gy. Mean dose, V_5Gy_ and V_17Gy_ of the ipsilateral lung were reduced by 0.2 Gy, 0.8% and 1.0% respectively with DMLC20‐IMRT. SS‐IMRT provided a slight gain of 0.1 Gy for the heart and 0.2 Gy on average doses to the contralateral lung and contralateral breast. These gains were small, averaging less than or equal to 1 Gy across various techniques.

Compared with SS‐IMRT, VMAT provided better D_98%_ and D_95%_ coverage of the PTV (1.1 Gy and 0.9 Gy) and much better conformity at 11.3%. VMAT only reduced V_10Gy_ (0.5%) and D_2%_ (1.6 Gy) of the LAD. For the other OARs, SS‐IMRT resulted in better reductions: 0.8 Gy versus 1.2 Gy on the average dose to the heart, 0.8 Gy on the average dose to the ipsilateral lung and a reduction in the dose to 5 Gy from 25.7% to 19.4%. For contralateral OARs, the mean dose to the lung was 0.3 Gy compared with 0.9 Gy, and the mean dose to the breast was 0.6 Gy compared with 1.2 Gy. The volume receiving 3 Gy for the contralateral breast increased from 0.6% to 1.7% in VMAT. The 5 Gy isodose was 1989.6 cm^3^ in SS‐IMRT compared with 2754.7 cm^3^ in VMAT.

In the final comparison between VMAT and DMLC20‐IMRT: there was no difference in PTV coverage. Conformity was better with VMAT, with a gain of 7.5%. However, the 20 Gy and 5 Gy isodoses were clearly reduced by 5.1% and 43.7% with DMLC20‐IMRT. For the OARs, the gains were statistically significant in favor of DMLC20‐IMRT: the mean dose to the heart decreased by 0.2 Gy, and the mean dose to the ipsilateral lung decreased by 0.9 Gy. The lung volume receiving 5 Gy was reduced from 25.7% to 18.6%. In contralateral organs, there were reductions of 0.4 Gy and 0.5 Gy in the mean dose to the breast and lung. Only 0.4% of the breast received 3 Gy compared with 1.7% in VMAT. There was no impact on LAD.

### 3.2 Radiobiological analysis

The results for TCP, NTCP and SCCP are given in Table 2. Analysis of TCP values demonstrates no significant differences across methods for the CTV, with all techniques presenting a TCP of 93.5% or 93.4%. For the PTV, TCP values are higher for VMAT and statistically significant with 92.9% versus 92.2% for DMLC20‐IMRT and 91.5% for SS‐IMRT. Additionally, TCP for DMLC10‐IMRT (92.0%) is significantly lower than that DMLC20‐IMRT.

The NTCP for the ipsilateral lung is lowest with VMAT (2.2%), compared to 3.7% for DMLC20‐IMRT and 4.1% for SS‐IMRT, marking a significant reduction with VMAT. For the heart, NTCP is consistently zero across all techniques, indicating no predicted pericarditis complication risk for this organ regardless of method.

When evaluating the risk of secondary cancer, the SCCP value for the contralateral breast is lowest with SS‐IMRT (0.4%), compared to 0.6% for both DMLC10/20‐IMRT, and 1.1% for VMAT (*p <* 0.05). This trend is consistent for the lungs overall, where SCCP is 1.6% for SS‐IMRT, 1.8% for DMLC10/20‐IMRT and 2.6% for VMAT, with statistical significance (*p <* 0.05).

### 3.3 Plan data analysis

Treatment plan data are presented in Table 3.

**TABLE 3:** Results of QA pre‐treatment, monitor units, complexity metrics with MCS and delivery time.

	SS‐IMRT			DMLC10‐IMRT			DMLC20‐IMRT			VMAT			*p*‐value	*p*‐value	*p*‐value	*p*‐value
	Mean	±	SD	Mean	±	SD	Mean	±	SD	Mean	±	SD	DMLC20 vs DMLC10	DMLC20 vs S&S	DMLC20 vs VMAT	VMAT vs S&S
GPR 3%/3 mm (%)	**99.8**	±	0.3	98.7	±	0.9	98.6	±	1.3	99.4	±	0.6	1.000	***<0.001***	***0.013***	***0.006***
MUs	**290.7**	±	18.6	530.7	±	82.7	518.2	±	70.9	487.7	±	53.0	0.114	***<0.001***	***0.021***	***<0.001***
MCS	**0.689**	±	0.044	0.337	±	0.052	0.339	±	0.043	NA	±	NA	0.648	***<0.001***	N/A	N/A
Delivery time (s)	95.4	±	6.6	83.3	±	12.5	**81.6**	±	9.9	141.7	±	28.6	0.161	***<0.001***	***<0.001***	***0.001***

*Note*: The optimal average values are highlighted in bold. *p*‐values less than 0.05 are highlighted in bold.

italics. A *p*‐value of less than 0.05 is considered to be significant.

Abbreviations: GPR, gamma passing rate; MCS, modulation complexity score; MUs, monitor units; N/A, not applicable.

There were no significant differences between the two DMLC variants. Between SS‐IMRT, DMLC20‐IMRT and VMAT, there was a significant drop in the QA result from 99.8% to 98.6% and 99.4%. The number of MUs increased from 290.7 (SS‐IMRT) to 518.2 (DMLC20‐IMRT) and 487.7 (VMAT). DMLC‐IMRT had greater modulation with the MCS, varying from 0.689 to 0.339. However, delivery time was shorter with DMLC‐IMRT at 81.6 seconds versus 95.4 seconds with SS‐IMRT. In VMAT, the delivery time is the highest with 141.7 seconds.

## 4 DISCUSSION

IMRT techniques produced clinically satisfactory results overall. Between the two DMLC‐IMRT configurations, DMLC20‐IMRT was generally superior to DMLC10‐IMRT across most parameters. Although the differences were small (< 0.2 Gy or 0.1%), statistical analysis supported selecting DMLC20‐IMRT.

When SS‐IMRT is inadequate, VMAT is the better alternative but DMLC20‐IMRT turns out to be an interesting option. Compared with SS‐IMRT, DMLC20‐IMRT improves PTV coverage and conformity, but slightly increases OAR doses and secondary cancer risk. TCP was higher with DMLC20‐IMRT than with SS‐IMRT (92.2% vs 91.2%) but lower than with VMAT (92.9%). SCCP values with DMLC20‐IMRT were intermediate for the contralateral breast and lungs (0.5% and 1.8%), exceeding SS‐IMRT (0.4% and 1.6%) but remaining below VMAT (0.9% and 2.6%). For the ipsilateral lung, DMLC20‐IMRT met the lowest constraint doses, whereas VMAT produced the lowest NTCP. IMRT also reduced low‐dose exposure: DMLC20‐IMRT lowered V_17Gy_ to 10.1% and mean lung dose to 4.8 Gy, versus 10.5% and 5.7 Gy with VMAT and 11.2% and 5.0 Gy with SS‐IMRT. However, VMAT reduced higher lung doses above 20 Gy; for example, V_30Gy_ was 7.0%, 6.5%, and 4.5% for SS‐IMRT, DMLC20‐IMRT, and VMAT, respectively (*p <* 0.05). Because V_30Gy_ is associated with pulmonary toxicity,^18^ the NTCP for radiation pneumonitis is slightly lower with VMAT. Its optimization allows better conformity of the isodose distribution to the convex contours of the PTV, reducing high‐dose exposure to the lung adjacent to the ribs and target volume. In contrast, tIMRT may reduce the risk of lung cancer because of its lower mean lung dose (4.8 Gy versus 5.7 Gy with cVMAT). Regarding cardiac outcomes, the NTCP for pericarditis remains equal to zero for all techniques. Mean heart doses are minimal, 0.8 Gy for SS‐IMRT, 1.0 Gy for DMLC‐IMRT and 1.2 Gy for cVMAT, with maximum average doses of 1.2 Gy, 1.3 Gy et 1.7 Gy for SS‐IMRT, DMLC20‐IMRT and cVMAT, respectively. Since no threshold exists for radiation‐induced cardiac risk,^1^, ^19^ minimizing heart dose is essential to avoid long‐term complications. All techniques achieve very low heart doses. In this context, SS‐IMRT offers the greatest benefit, followed by DMLC‐IMRT, with VMAT showing slightly higher cardiac doses. Although the differences in TCP, NTCP, and SCCP were statistically significant, their absolute magnitudes were small. Given the uncertainty of radiobiological models and the limited sample size, these results should be interpreted with caution, and their clinical relevance is likely limited.

cVMAT showed the best conformity (CI 0.83 vs 0.77 for DMLC20‐IMRT and 0.74 for SS‐IMRT) but delivered higher posterior doses. While the 20 Gy isodose was similar across techniques, SS/DMLC20‐IMRT reduced the 5 Gy volume (about 2000 cm^3^ vs 2800 cm^3^ with VMAT). Among the 20 breasts evaluated, TCP deviation exceeding 1% between VMAT and DMLC20‐IMRT was observed in 5 instances, as opposed to 8 instances with SS‐IMRT. Three factors can explain these differences: breast morphology, as marked lung convexity increases lung volume in BEV of tangential beams; LAD proximity to the tangential field edges, which can reduce PTV coverage; and larger breast size, which increases perimeter and may further reduce PTV coverage. When breast diameter was at least 17 cm, especially with these features, VMAT provided better PTV coverage and higher TCP. Based on a sample of 20 treatment plans, VMAT appears to be superior for breasts with a diameter greater than 20 cm. This finding warrants confirmation with a larger number of cases. However, with a 6 MV two beam configuration, in order to achieve 100% of the prescribed dose at the center, the entrance doses will be high, which will significantly degrade homogeneity in PTV. An energy level higher than 10 MV would be required, but this results in a reduction in dose in the first few millimeters of the breast tissue, which could be clinically detrimental and is not used by our institution. DMLC20‐IMRT improved coverage over SS‐IMRT. In the case shown in Figure 1 (17 cm breast diameter, LAD 4 mm from the tangential field edges, and 4 cm of left lung visible on BEV), VMAT outperformed IMRT, with TCP gains of 1.8% over DMLC20‐IMRT and 6.1% over SS‐IMRT. DMLC20‐IMRT remained close to VMAT. None of the three techniques reduced the left lung volume receiving 17 Gy below 14%. Above 16 Gy, VMAT lowered lung dose slightly but increased doses to other OARs.

The literature shows that VMAT generally improves target coverage, although this advantage is reduced with DMLC20‐IMRT, which also lowers OAR doses. Table 4 summarizes comparative studies^5^, ^6^, ^7^, ^8^, ^9^, ^10^, ^11^, ^12^ involving tIMRT, multi‐beam IMRT, cVMAT, tVMAT, 3DRCT, field‐in‐field, and Tomotherapy. Sakka et al.^5^ reported better LAD dosimetry with IMRT, whereas Koivumäki et al.^6^ found that VMAT improves coverage and homogeneity but slightly increases dose to contralateral OARs. Wu et al.^12^ also reported better planning performance with VMAT, though their IMRT technique used five non‐tangential beams. Comparisons across studies remain limited by differences in dose prescriptions, constraints, CI definitions, and beam arrangements. Overall, cVMAT tends to provide more conformal and homogeneous plans than IMRT, but at the cost of higher doses to contralateral OARs. tVMAT shortens treatment compared with cVMAT while maintaining similar coverage, whereas cVMAT better spares the heart, LAD, and ipsilateral lung. Our study therefore aims to identify an approach between SS‐IMRT and cVMAT when tVMAT is insufficient.

**TABLE 4:** Studies for the left breast.

		Prescription	Technique	Linac & MLC	TPS	PTV	PTV	HI	CI	Ipsilateral lung				Heart	LAD	Contralateral	
										Dmean (Gy)	V_5Gy_ (%)	V_20Gy_ (%)	V_17Gy_ (%)	D_mean_ (Gy)	D_mean_ (Gy)	Lung D_mean_	Breast (Gy)
2017	Sakka et al.^5^	50.4 Gy	IMRT 5 ‐ 7 beams		Pinnacle	V_95%_ > 95%				10.4		14.2		3.0	5.8	2.0	1.8
			cVMAT 2 arcs 178° to 300°		Eclipse	V_95%_ > 95%				9.9		14.0		4.0	7.3	3.5	3.3
2018	Koivumäki et al.^6^	40.05 Gy	Field‐in‐field 2 tangential beams	Varian Ix MLC 120 millenium	Eclipse	V_95%_ = 96.6%	D_1cm3 _= 107.5%	0.11		6.7	25.9	14.2		1.3	6.1	0.1	0.1
			tVMAT 2 arcs 45°‐55°	Varian Ix MLC 120 millenium	Eclipse	V_95%_ = 98.3%	D_1cm3 _= 105.7%	0.08		6.6	30.2	12.6		1.6	4.2	0.6	0.9
			4‐6 tangential beams	Accuray TomoDirect	Accuray	V_95%_ = 98.3%	D_1cm3 _= 105.7%	0.08		5.8	24.9	11.3		1.3	4.7	0.2	0.3
			cVMAT 2 arcs 259° to 173°	TrueBeam MLC 120 millenium	Eclipse	V_95%_ = 97.7%	D_1cm3 _= 106.0%	0.01		6.5	36.4	9.1		1.9	4.0	0.3	0.6
2019	Testolin et al.^7^	42.4 and 50 Gy	tIMRT		Eclipse	V_95%_ = 96.1%				4.9		8.5		0.9	4.3	0.1	0.1
2019	Karpf et al.^8^	50.4 Gy	IMRT 6 ‐ 8 beams between 300° et 175°		Pinnacle	V_95%_ > 95%				10.4				3.0	5.8		
			tVMAT 4 partial arcs bettween 200° to 179°		Eclipse	V_95%_ > 95%				9.9				4.0	7.3		
2021	Gaál et al.^9^	50 Gy	Field‐in‐field 2 tangential beams	Varian TrueBeam STx	Eclipse	V_95%_ > 90%		0.10		6.4		10.7		1.4	4.6		0.5
2022	Redapi et al.^10^	42.56 Gy	tIMRT‐DMLC	Elekta Infinty MLC Agility	Monaco	D_98%_ = 40.5 Gy	V_105%_ = 1.0%	0.09		7.3	27.5	14.7		2.1		0.3	0.7
			tIMRT‐SS	Elekta Infinty MLC Agility	Monaco	D_98%_ = 40.6 Gy	V_105%_ = 3.5%	0.10		7.2	26.0	14.5		2.0		0.4	0.7
			tVMAT 2 arcs 100° to 160° and 90° to 350°	Elekta Infinty MLC Agility	Monaco	D_98%_ = 40.6 Gy	V_105%_ = 1.5%	0.09		5.2	21.3	8.6		1.6		0.7	1.1
2022	S Nair et al.^11^	42.56 Gy	3DCRT		Monaco	V_95%_ = 95.8%			0.68	8.6		17.7		2.2	5.5	0.3	
			IMRT 5 beams (330°, 10°, 50°, 100°, 150°)		Monaco	V_95%_ = 98%			0.76	6.6		16.0		7.5	16.5	5.6	
			cVMAT 2 arcs of 200°		Monaco	V_95%_ = 98.1%			0.85	6.4		15.1		5.8	15.0	4.8	
2024	Wu et al.^12^	50 Gy	IMRT 5 beams (315°, 300°, 105°, 145°, 120°)	Varian VitalBeam	Eclipse	V_95%_ = 99.2%	D_2%_ = 53.9 Gy	0.10	0.80	6.5	25.1	11.5		1.9	9.2	0.1	0.7
			tVMAT 4 arcs (300° to 0° and 90° to 145°)	Varian VitalBeam	Eclipse	V_95%_ = 99.2%	D_2%_ = 54.2 Gy	0.10	0.80	6.1	24.8	10.7		1.7	9.0	0.1	1.0
2026	This study	40.05 Gy	tIMRT‐SS	Elekta Infinty MLC Agility	RayStation	V_95%_ = 94.3%	D_2%_ = 41.8 Gy	0.15	0.74	5.0	19.4		11.2	0.8	2.2	0.3	0.6
			tDMLC20‐IMRT	Elekta Infinty MLC Agility	RayStation	V_95%_ = 96.3%	D_2%_ = 41.6 Gy	0.11	0.77	4.8	18.6		10.2	1.0	2.1	0.5	0.7
			cVMAT 2 arcs 170° to 300°	Elekta Infinty MLC Agility	RayStation	V_95%_ = 96.6%	D_2%_ = 41.5 Gy	0.11	0.83	5.7	25.7		10.5	1.2	2.2	0.9	1.2

Abbreviations: CI, conformal index; Dmean, median dose; Dx, dose to x% of the volume; HI, homogeneity index; LAD, left anterior descending coronary artery; PTV, planning target volume; TPS, treatment planning system; Vx, % volume receiving x Gy.

After accounting for prescription dose differences, DMLC20‐IMRT yielded the lowest mean doses to the heart, LAD, and both lungs while maintaining adequate PTV coverage. Both DMLC20‐IMRT and cVMAT demonstrate slightly reduced homogeneity (HI = 0.11) whereas other studies report HI values below or equal to 0.10, reflecting a trade‐off between homogeneity and OAR sparing. Internal tVMAT testing increased ipsilateral lung and LAD doses to levels reported in previous studies. Because tVMAT cannot use the dual arc feature, its optimization is less effective, leading us to select cVMAT for breast treatments. To create tVMAT plans that met our criteria, the position of the isocenter must be adapted from our usual position. Two important points are used to determine the position of the isocenter. First, the position must avoid collision with the table and/or the patient over the full 360°. A weekly CBCT is performed in FB to verify the absence of morphological changes. Secondly, the isocenter is positioned so that the MLC covers a sufficient volume, considering possible breast swelling.^17^ Thus, the isocenter is in the middle of the sternum and the table height is adjusted to avoid collision when the gantry is in the posterior position. Under these internally chosen clinical conditions, tVMAT demonstrates significantly lower effectiveness than the cVMAT approach. Placement of the isocenter within the PTV or in the upper part of the lung in the median plane of the PTV, as referenced in prior,^4^, ^7^, ^8^, ^9^, ^10^, ^11^, ^12^ yields notable improvements in the tVMAT plan. Nevertheless, in our institution the objective remains to establish a uniform position of the isocenter for the treatment of all breasts (with or without lymph node involvement) which complies with CBCT requirements and mitigates risks of table or patient collision. IGRT complements SGRT for setup verification^15^ using 2D kV or 2D MV, and CBCT. It could be conducted during DIBH, with particular consideration given to CBCT due to its longer acquisition time compared to basic 2D MV or kV imaging. Modern linear accelerators facilitate monitoring during CBCT procedures. Alternatively, CBCTs may be acquired in separate DIBHs; however, this approach is time‐intensive and may pose challenges for the patient, especially prior to treatment delivery. Partial CBCT acquisitions (approximately 25 seconds over 200°) are feasible but restrict the field of view (FOV), limiting complete breast visualization. Monitoring a partial CBCT scan is difficult because the kV panel and X‐ray tube obstruct the cameras. Since partial CBCT is difficult to implement in DIBH, an interesting alternative is to acquire an MV image using DIBH in tIMRT. The isocenter positioning is determined by the imaging requirements outlined in the protocol for IGRT and SGRT. For 2D MV, kV, or partial CBCT imaging, the isocenter can typically be placed within the median plane of the left breast, with table height adjusted to prevent collisions. If a full CBCT is necessary, possible collisions involving the patient and table must be evaluated. However, cVMAT could obstruct the SGRT camera twice between 20° and 60°, which may disrupt surface tracking during the DIBH treatment. In our workflow, both DIBH and FB plans are generated: FB is chosen when DIBH offers no dosimetric benefit, whereas tIMRT is preferred when DIBH is superior to avoid SGRT issues. Thus, when SS‐IMRT is insufficient, DMLC‐IMRT is a practical alternative to VMAT because it improves outcomes while avoiding radiotherapy departmental workflow complications from camera obstruction and angle adaptation. For VMAT treatments, RTTs will assess potential camera obstruction during the first treatment session and adjust the ROI if necessary. If the obstruction cannot be resolved, a new treatment plan will be generated with the problematic beam angles excluded. With the Advanced Camera Optimization (ACO) for the AlignRT system, obstructions are reduced (0.1 mm on a phantom), but in routine clinical practice, it has been observed that obstructions persist, although their number is significantly reduced during treatment, they remain present during CBCT and are not visible with 2D MV.

A major difference between IMRT and VMAT is delivery time. DMLC‐IMRT reduces beam time by 13.8 s versus SS‐IMRT (95.4 s) and remains much faster than VMAT (81.6 s vs. 141.7 s). This shorter duration may ease breath hold effort. Because not all patients are eligible^7^, ^9^, ^20^ for DIBH (between 10% and 15% of cases involving apnea lasting less than 20 or 30 seconds and 39% of cases for poor reproducibility or poor breath‐hold stability), DMLC may make treatment feasible in some cases where VMAT would be too complex. Because apnea duration and frequency are not restricted, the shorter DMLC delivery time may help more patients benefit from DIBH while reducing fatigue. However, this was not demonstrated in the present study.

Higher DMLC modulation increased MUs, producing a slightly less precise but still acceptable agreement between measurement and calculation under institutional criteria. QA testing showed no issues, as the maximum remained below 600 MUs for a 2.67 Gy fraction. These dosimetric results and acceptable agreement supported validation of the DMLC mode with 20 CPs.

## 5 CONCLUSION

Three significant differences may impact the selection of DIBH treatment techniques for the left breast. Firstly, although these results have already been published, VMAT offers superior PTV conformity, albeit with a marginally increased dose to the OARs. VMAT should be considered when two SS‐IMRT or DMLC‐IMRT tangential beams do not adequately meet the PTV coverage requirements, particularly for atypical shapes, large breasts or the LAD position. Secondly, the X‐ray delivery durations for IMRT (95.4 seconds for SS‐IMRT or 81.6 seconds for DMLC‐IMRT) are notably shorter than for VMAT (141.7 seconds), thereby enabling broader DIBH accessibility and decreasing patient fatigue and stress during treatment sessions. Thirdly, interruptions due to SGRT reconstruction issues when the gantry, imaging panel, and X‐ray tube obstructs a camera are inconsequential in IMRT but can pose challenges in VMAT. In this context, DMLC‐IMRT represents a compelling alternative. The choice of treatment technique should be made by each institution according to its available equipment, while considering the dosimetric characteristics of each technique and its suitability for the patient's anatomy. These three points were identified in a cohort of just 20 patients at a single center equipped with one TPS. A multicenter study involving a larger sample size and different equipment could, in the future, provide more robust conclusions regarding the clinical impact of the various methods.

## AUTHOR CONTRIBUTIONS

Julien Rolland: conceptualization, data curation, methodology, software and writing–original draft. Stéphane Payan, Pierre Fau, Hugues Mailleux and Agnès Tallet: final manuscript review.

## CONFLICT OF INTEREST STATEMENT

The authors declare no conflicts of interest

## FUNDING INFORMATION

This study did not receive any specific grant from funding agencies in the public, commercial, or not‐for‐profit sectors.

## ETHICS STATEMENT

Ethics approval were waived for this study because no patients’ data were reported

## Supporting information



Supporting Information 1: acm270761‐sup‐0001‐SuppMat.docx.

Supporting Information 2: acm270761‐sup‐0002‐SuppMat.pdf.

Supporting Information 3: acm270761‐sup‐0003‐SuppMat.pdf.

## Data Availability

The data that support the findings of this study are available from the corresponding author upon reasonable request.
