# Supplementary material for: Dosimetric and radiobiological evaluations as well as technical aspects of left breast treatment employing DIBH with SGRT in radiotherapy with IMRT step and shoot, IMRT Dynamic MLC and VMAT
Source: J Appl Clin Med Phys. 2026 Aug 25;27(9):e70761. doi: 10.1002/acm2.70761 (PMC13504233; doi:10.1002/acm2.70761)
Supplement: Supplementary file 1 [file ACM2-27-e70761-s001.pdf]

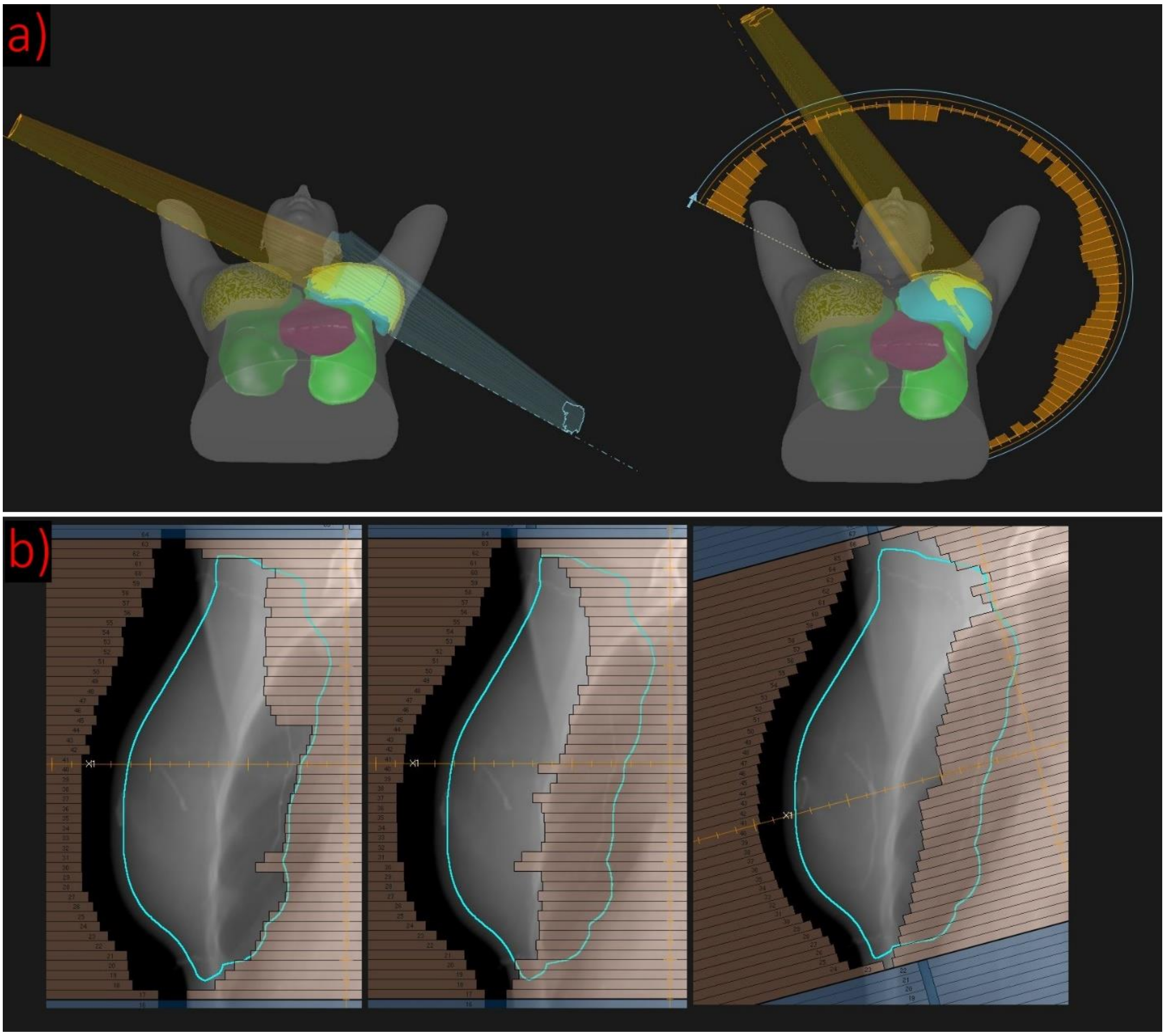

FIGURE S1 a) tIMRT and cVMAT beams arrangement; b) geometric shape of the MLC to cover any volume or swelling outside the breast, SS-IMRT, DMLC-IMRT and VMAT respectively.
