# Supplementary material for: Dosimetric and radiobiological evaluations as well as technical aspects of left breast treatment employing DIBH with SGRT in radiotherapy with IMRT step and shoot, IMRT Dynamic MLC and VMAT
Source: J Appl Clin Med Phys. 2026 Aug 25;27(9):e70761. doi: 10.1002/acm2.70761 (PMC13504233; doi:10.1002/acm2.70761)
Supplement: Supplementary file 2 [file ACM2-27-e70761-s002.pdf]

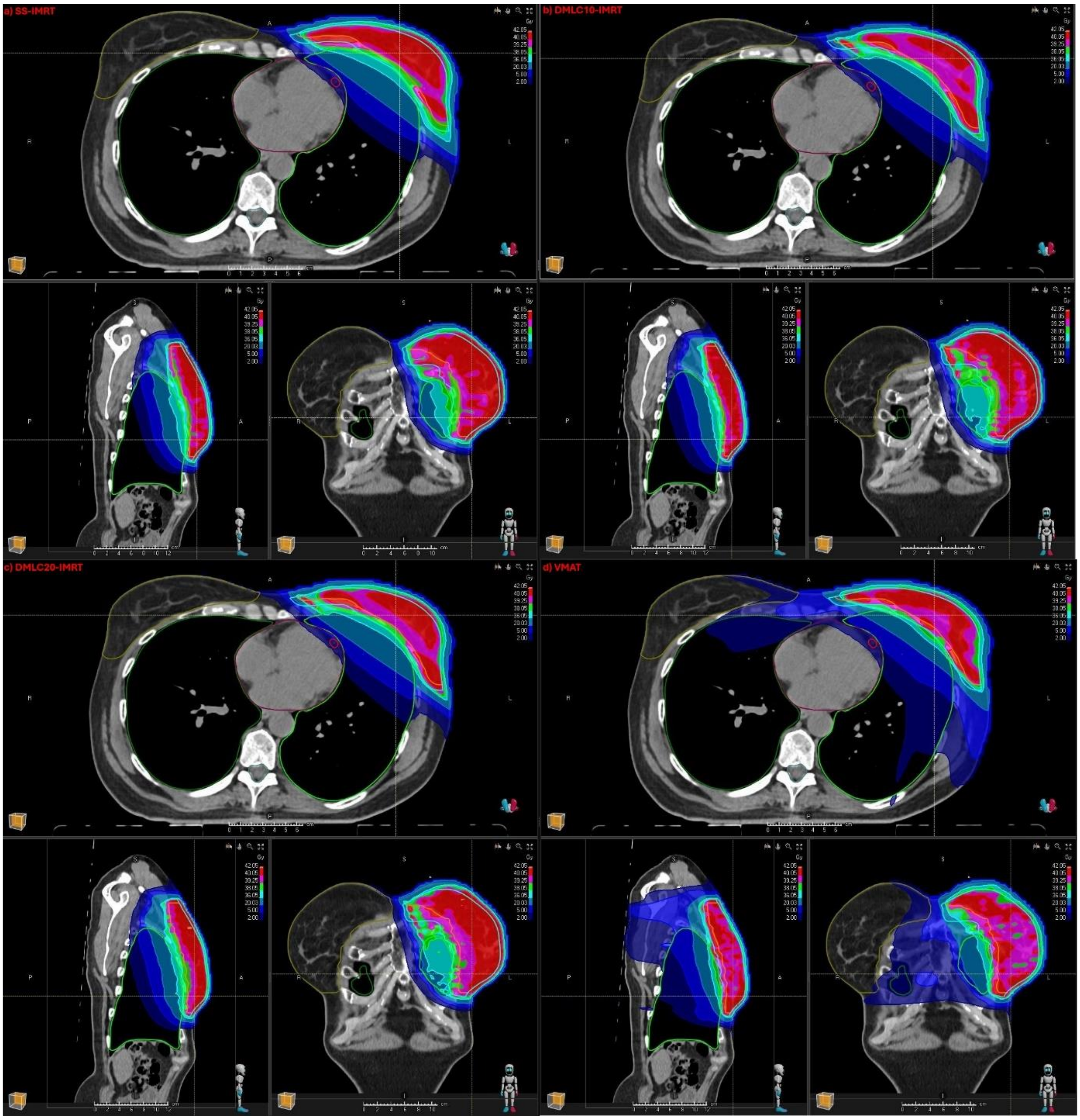

FIGURE S2 The typical dose distributions in 3 axial plans for one patient and all methodologies: a) SS-IMRT; b) DMLC10-IMRT; c) DMLC20-IMRT; d) VMAT. Visible and contoured volumes are: PTV in light blue, CTV in orange, LAD in pink, heart in purple, ipsilateral lung in light green, contralateral lung in dark green, contralateral breast in kaki green.
