# Supplementary material for: Dosimetric and radiobiological evaluations as well as technical aspects of left breast treatment employing DIBH with SGRT in radiotherapy with IMRT step and shoot, IMRT Dynamic MLC and VMAT
Source: J Appl Clin Med Phys. 2026 Aug 25;27(9):e70761. doi: 10.1002/acm2.70761 (PMC13504233; doi:10.1002/acm2.70761)
Supplement: Supplementary file 3 [file ACM2-27-e70761-s003.docx]

**TABLE S1** Radiobiological parameters.

| **Parameter** | **Volume** | **α/β** | **a** | **γ_50_** | **TCD_50_(Gy)** | **TD_50_(Gy)** | **m** | **n** | **End point** | **In_org_ (%/Gy)** | **δ_org_(Gy^-1^)** |
| --- | --- | --- | --- | --- | --- | --- | --- | --- | --- | --- | --- |
| TCP^1-3^ | CTV, PTV | 4 | -7.2 | 1.3 | 30.86 |  |  |  |  |  |  |
| NTCP^4-6^ | Heart | 3 |  |  |  | 48 | 0.1 | 0.35 | Pericarditis |  |  |
| NTCP | Ispsilateral lung | 3 |  |  |  | 30.8 | 0.18 | 0.87 | Pneumonitis |  |  |
| SCCP^7-8^ | Contralateral breast |  |  |  |  |  |  |  |  | 0.78 | 0.085 |
| SCCP | Lung(s) |  |  |  |  |  |  |  |  | 8.27 | 0.129 |

Abbreviations: TCP, tumor control probability; NCTP, normal tissue complication probability; SCCP, second cancer complication probability.

Note: α/β intrinsic radiosensitivity of a specific tissue; a is the defined specific parameter-tumor tis­sue describing the dose-volume effect; γ_50_ is the slope of the dose-response curve in TCP; TCD_50_(Gy) : the uniform dose administered to the entire organ that results in 50 % complication risk; TD_50_(Gy) : Dose associated with 50 % complication probability ; m is the curved slope; n is a parameter describing the importance of the volume effect; In_org_ :Organ-specific absolute cancer ; δ_org_ cellular radio-sensitivity incidence.

References:

| **1.** | Niemierko A. Reporting and analyzing dose distributions: a concept of equivalent uniform dose. Med Phys. 1997 Jan;24(1):103-10. doi: 10.1118/1.598063. PMID: 9029544. |
| --- | --- |
| **2.** | Niemierko A, Goitein M. Implementation of a model for estimating tumor control probability for an inhomogeneously irradiated tumor. Radiother Oncol 1993; 29: 140–7. doi: 10.1016/0167-8140(93)90239-5 |
| **3.** | Niemierko, A. (1999) A Generalized Concept of Equivalent Uniform Dose (EUD). Medical Physics, 26, 1100. |
| **4.** | Kutcher GJ, Burman C. Calculation of complication probability factors for non-uniform normal tissue irradiation: the effective volume method. Int J Radiat Oncol Biol Phys. 1989 Jun;16(6):1623-30. doi: 10.1016/0360-3016(89)90972-3. PMID: 2722599. |
| **5.** | Lyman JT, Wolbarst AB. Optimization of radiation therapy, III: A method of assessing complication probabilities from dose-volume histograms. Int J Radiat Oncol Biol Phys. 1987 Jan;13(1):103-9. doi: 10.1016/0360-3016(87)90266-5. PMID: 3804804. |
| **6.** | Burman C, Kutcher GJ, Emami B, Goitein M. Fitting of normal tissue tolerance data to an analytic function. Int J Radiat Oncol Biol Phys. 1991 May 15;21(1):123-35. doi: 10.1016/0360-3016(91)90172-z. PMID: 2032883. |
| **7.** | Schneider U, Zwahlen D, Ross D, Kaser-Hotz B. Estimation of radiation-induced cancer from three-dimensional dose distributions: Concept of organ equivalent dose. Int J Radiat Oncol Biol Phys. 2005 Apr 1;61(5):1510-5. doi: 10.1016/j.ijrobp.2004.12.040. PMID: 15817357. |
| **8.** | Schneider U, Kaser-Hotz B. A simple dose-response relationship for modeling secondary cancer incidence after radiotherapy. Z Med Phys. 2005;15(1):31-7. doi: 10.1078/0939-3889-00242. PMID: 15830782. |
